# Mesenchymal Stem Cells Alleviate Post-resuscitation Cardiac and Cerebral Injuries by Inhibiting Cell Pyroptosis and Ferroptosis in a Swine Model of Cardiac Arrest

**DOI:** 10.3389/fphar.2021.793829

**Published:** 2021-12-09

**Authors:** Jiefeng Xu, Minhai Zhang, Fei Liu, Lin Shi, Xiangkang Jiang, Chuang Chen, Jiangang Wang, Mengyuan Diao, Zafar Ullah Khan, Mao Zhang

**Affiliations:** ^1^ Department of Emergency Medicine, Second Affiliated Hospital, Zhejiang University School of Medicine, Hangzhou, China; ^2^ Key Laboratory of The Diagnosis and Treatment of Severe Trauma and Burn of Zhejiang Province, Hangzhou, China; ^3^ Zhejiang Province Clinical Research Center for Emergency and Critical Care Medicine, Hangzhou, China; ^4^ Department of Emergency Medicine, Zhejiang Hospital, Hangzhou, China; ^5^ Hangzhou Emergency Medical Center, Hangzhou, China; ^6^ Department of Intensive Care Medicine, Affiliated Hangzhou First People’s Hospital, Zhejiang University School of Medicine, Hangzhou, China

**Keywords:** cardiac arrest, cardiopulmonary resuscitation, post-resuscitation, cardiac injury, cerebral injury, mesenchymal stem cell, pyroptosis, ferroptosis

## Abstract

Following cardiopulmonary resuscitation (CPR), the ensuing cardiac and cerebral injuries contribute to the poor outcome of cardiac arrest (CA) victims, in which the pathogenetic process is possibly driven by cell pyroptosis and ferroptosis. Mesenchymal stem cells (MSCs) have been shown to be a promising strategy for post-resuscitation cardiac and cerebral protection in rat, but its effectiveness in the clinically relevant swine model and the potential protective mechanism remain unknown. The present study was designed to investigate whether MSCs administration could alleviate post-resuscitation cardiac and cerebral injuries through the inhibition of cell pyroptosis and ferroptosis in swine. Twenty-four male domestic swine were randomly divided into three groups: sham, CPR, and MSC. A dose of 2.5×10^6^/kg of MSCs derived from human embryonic stem cells was intravenously infused at 1.5, and 3 days prior to CA. The animal model was established by 8 min of CA and then 8 min of CPR. After resuscitation, cardiac, cerebral function and injury biomarkers were regularly evaluated for a total of 24 h. At 24 h post-resuscitation, pyroptosis-related proteins (NLRP3, ASC, cleaved caspase-1, GSDMD), proinflammatory cytokines (IL-1β, IL-18), ferroptosis-related proteins (ACSL4, GPX4) and iron deposition in the heart, cortex and hippocampus were measured. Consequently, significantly greater cardiac, cerebral dysfunction and injuries after resuscitation were observed in the CPR and MSC groups compared with the sham group. However, the severity of cardiac and cerebral damage were significantly milder in the MSC group than in the CPR group. In addition, the expression levels of NLRP3, ASC, cleaved caspase-1, GSDMD and ACSL4, the contents of IL-1β and IL-18, and the level of iron deposition were significantly higher while the expression level of GPX4 was significantly lower in the heart, cortex and hippocampus in all resuscitated animals compared with the sham group. Nevertheless, MSCs administration significantly decreased post-resuscitation cardiac, cerebral pyroptosis and ferroptosis compared to the CPR group. Our results showed that the administration of MSCs significantly alleviated post-resuscitation cardiac and cerebral injuries in swine, in which the protective effects were related to the inhibition of cell pyroptosis and ferroptosis.

## Introduction

After obtaining successful cardiopulmonary resuscitation (CPR) from cardiac arrest (CA) events, most patients will die of post-resuscitation cardiac and cerebral injuries in the following hospitalization (Laver et al., 2004; Neumar et al., 2008). However, cardiac and cerebral pathogenetic mechanism after resuscitation and their effective therapeutic target remain to be investigated (Burstein and Jentzer, 2020). Recently, a series of studies have confirmed that cell pyroptosis and ferroptosis known as two novel forms of regulated cell death play important roles in the pathogenetic process of regional cardiac and cerebral ischemia/reperfusion (IR) injury (Datta et al., 2020; Lu et al., 2021). In the setting of CA and resuscitation, two investigations have preliminarily manifested that cell pyroptosis was involved in the pathogenesis of cardiac and cerebral injuries after resuscitation and could become a new therapeutic target (Chang et al., 2020; He et al., 2020). Currently, it is necessary to further confirm the occurrence of cell pyroptosis and ferroptosis in the heart and brain after resuscitation and their regulatory strategies in CA models.

Mesenchymal stem cells (MSCs), derived from bone marrow, placenta, adipose or other tissues, have been proven to be a promising therapy against regional cardiac and cerebral IR injury in diverse of studies (Yan et al., 2019; Li et al., 2021). In the small-animal models of CA and resuscitation, MSCs administered before CA or after resuscitation have also shown its potent protection for post-resuscitation cardiac, cerebral dysfunction and injuries. Earlier, two studies demonstrated that MSCs pretreatment and posttreatment significantly improved post-resuscitation cardiac and cerebral function in rats, respectively (Wang et al., 2008; Wang et al., 2009). Recently, another one study demonstrated that the administration of MSCs significantly ameliorated post-resuscitation brain damage, and promoted its angiogenesis and neurological recovery through enhancing the expression of brain-derived neurotrophic factor and vascular endothelial growth factor in rats (Zhou et al., 2017). However, the effectiveness of MSCs administration in a clinically relevant, large-animal model of CA and resuscitation and its potential protective mechanism require further investigations.

In the present study, we attempted to investigate the effects of MSCs administration on post-resuscitation cardiac and cerebral outcomes in a clinically relevant swine model. We hypothesized that the administration of MSCs would alleviate post-resuscitation cardiac, cerebral dysfunction and injuries in swine. In addition, cell pyroptosis and ferroptosis would occur in the heart and brain after resuscitation; however, both of them would be inhibited in animals treated with MSCs.

## Materials and Methods

### Mesenchymal Stem Cells

**FIGURE 1:**
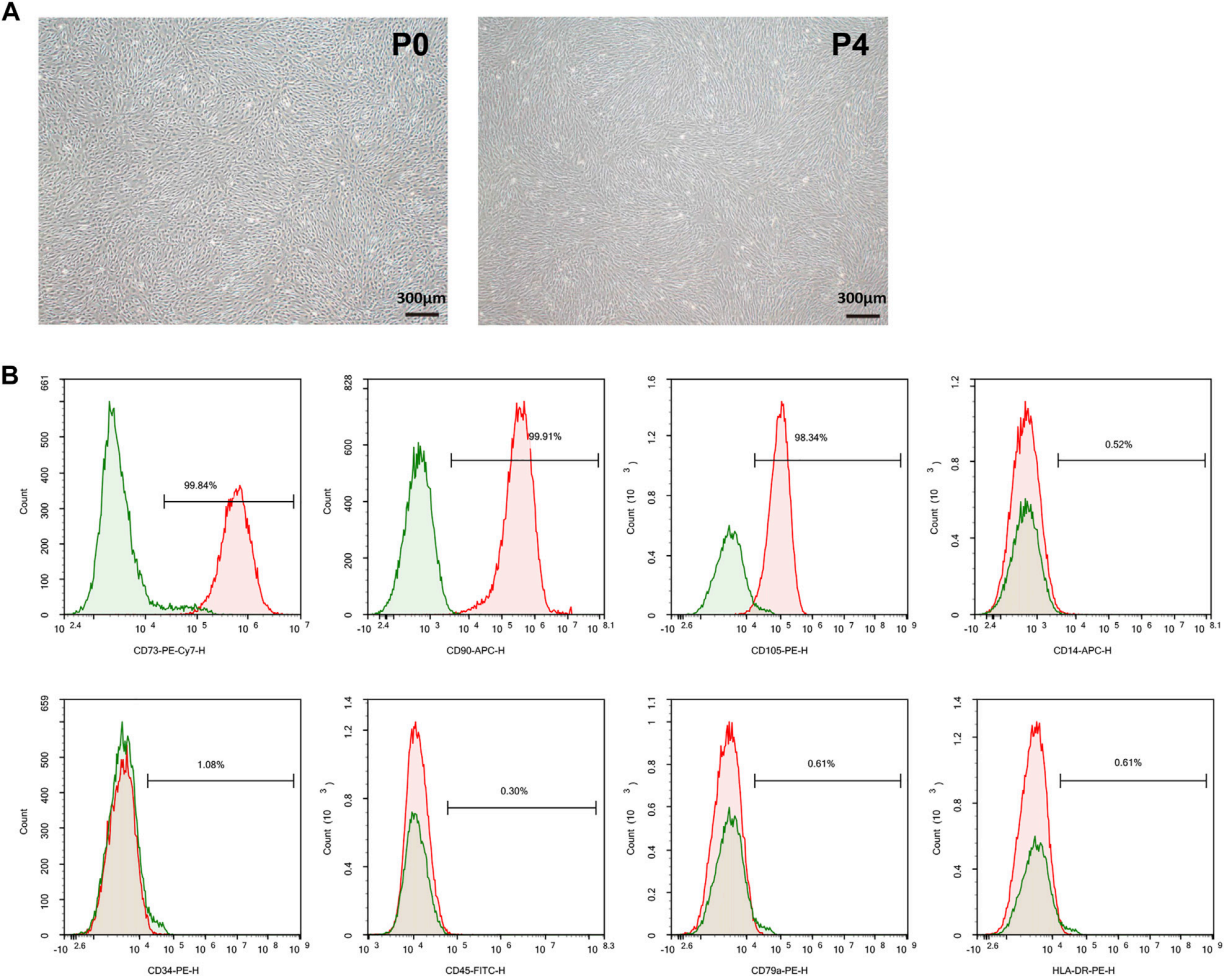
Characterization of hESC-derived MSCs **(A)** Representative micrographs of morphological characterization of MSCs at passage 0 (P0) and passage 4 (P4) **(B)** Flow cytometry analysis for specific surface antigen markers of MSCs at P4. hESC, human embryonic stem cell; MSC, mesenchymal stem cell.

MSCs were differentiated from human embryonic stem cells (hESCs) using a two-step process involving embryoid body (EB) formation, followed by outgrowth of EBs over plates. Briefly, H9-hESCs colonies (YuanSheng Biotech Corporation, Hangzhou, China) were dissociated into small clumps after 3 min of incubation with TrypLE express and then transferred to ultralow-attachment plates in E8 media (Gibco, Carlsbad, United States). After 7 days, EBs were harvested and plated onto plates in MSC induction medium including Dulbecco’s modified Eagle medium (high glucose), 10% fetal bovine serum and 1 mM l-glutamine. After 2 weeks, the EB outgrowths were sub-cultured using TrypLE Express. These were termed hESC-derived MSCs and were designated as passage 0 (P0). The differentiated hESCs attained homogenous population of spindle-shaped cells. Passage 4 (P4) hESC-derived MSCs were used in the following animal experiment. The morphology of MSCs at P0 and P4 was shown in Figure 1A.

According to the Mesenchymal and Tissue Stem Cell Committee of the International Society for Cellular Therapy, the minimum criteria for MSC identification is >95% cell population positive for CD73, CD90 and CD105, and <2% negative for CD11b or CD14, CD19 or CD79a, CD34, CD45 and HLA class II (Horwitz et al., 2005). In this study, the phenotype identification of MSCs was performed using flow cytometry (BD Biosciences, San Jose, United States) and the following monoclonal antibodies: anti-CD73-PE-Cy7, anti-CD90-APC, anti-CD105-PE, anti-CD14-APC, anti-CD34-PE, anti-CD45-FITC, CD79a-PE, and HLA-DR-PE. All the antibodies were purchased from BD Pharmingen (Shanghai, China). Briefly, the cells were dissociated and suspended in FACS buffer (1x PBS/0.5% BSA), and then incubated with the above-mentioned fluorescent conjugated antibodies for 20 min at 4°C. After antibody labeling, data was acquired using Agilent NovoCyte and analyzed using NovoExpress. The results of MSCs identification were shown in Figure 1B, in which the percentages of CD73, CD90 and CD105 positive cells were 99.84, 99.91, and 98.35%, respectively; and meanwhile CD14, CD34, CD45, CD79a and HLA-DR were all negative on MSCs (all <1.1%).

### Animals

Healthy male white domestic swine, aged 4–6 m, weighing 39 ± 2 kg, were purchased from Shanghai Jiagan Biotechnology Inc (Shanghai, China). All animals received humane care in compliance with the “Principles of Laboratory Animal Care” formulated by the National Society for Medical Research and the Guide for the Care and Use of Laboratory Animals prepared by the Institute of Laboratory Animal Resources. The animals were fed in the following conditions including standard atmospheric pressure, 12/12 h light/dark cycle, 20–25°C of room temperature, 60–80% of humidity, spontaneous water intake, and regular feeding, cleaning and disinfection. The whole experimental protocols were approved by the Institutional Animal Care and Use Committee of the Second Affiliated Hospital, Zhejiang University School of Medicine.

### Anesthesia and Surgical Preparation

The animals were fasted but allowed to drink water the night before the experiment. After that, the anesthesia was initially performed by an intramuscular injection of midazolam (0.4–0.5 mg/kg) followed by an ear vein injection of propofol (2 mg/kg), and then maintained by a continuous infusion of propofol (4 mg/kg/h). Subsequently, the animals were intubated using a cuffed endotracheal tube and then ventilated with an emergency and transport ventilator (Oxylog 3000 plus, Drager, Luebeck, Germany) with a tidal volume of 10 ml/kg, peak flow of 40 L/min and FiO_2_ of 0.21. End-tidal CO_2_ was monitored with a M Series Monitor Defibrillator (ZOLL Medical Corporation, Chelmsford, United States), and maintained at a normal physiological level of 35–40 mm Hg through the adjustment of respiratory frequency. The conventional lead II electrocardiogram was monitored by applying four adhesive electrodes to the bilateral upper-and-lower limbs.

For the measurements of aortic and right atrial pressures and the collection of arterial and venous blood samples, two fluid-filled 7 Fr thermodilution-tipped catheters were advanced from the right femoral artery and vein into the thoracic aorta and right atrium, respectively. For the measurements of stroke volume (SV) and global ejection fraction (GEF), a 4 Fr thermistor-tipped arterial catheter was inserted into the left femoral artery, another 7 Fr central venous catheter was inserted into the right internal jugular vein, and then both of them were connected to the PiCCO Monitor system (PiCCOplus, Pulsion Medical Systems, Munich, Germany). All arterial and venous catheters were intermittently flushed with saline containing 5 IU bovine heparin per ml. For the induction of ventricular fibrillation (VF), a 5 F pacing catheter was inserted from the right external jugular vein into the right ventricle. Rectal temperature was monitored with a thermal probe and maintained at a normal range of 38.0 ± 0.5°C with the aid of the Blanketrol III Hyper-Hypothermia System (Cincinnati Sub-Zero, Cincinnati, United States).

### Experimental Procedures

The animals were randomized with the Sealed Envelope Method into one of the three groups: sham group (n = 6), CPR group (n = 10), and MSC group (n = 8). In the MSC group, MSCs were administered via an ear vein at 1.5, and 3 days prior to CA, in which a dose of 2.5×10^6^/kg cells diluted in 200 ml of normal saline was infused within 40 min every time. In the sham and CPR groups, the same volume of normal saline was administered at the same time.

After the surgical preparation were completed, the animals were stabilized for 15 min and then baseline measurements were obtained. Subsequently, the animal model was established by 8 min of CA and then 8 min of CPR. In the sham group, the animals only underwent the surgical preparation without experiencing CA and resuscitation. In the CPR and MSC groups, VF was induced by 1 mA alternating current delivered to right ventricular endocardium. Once VF was confirmed, mechanical ventilation was stopped and the pacing catheter was withdrawn. After 8 min of untreated VF, CPR was performed by continuous chest compression and mechanical ventilation. Chest compression was provided by two professional CPR providers and their compression quality was monitored by a CPR feedback device (PlamCPR, Sunlife, Shanghai, China) to guarantee a compression depth of 50–60 mm at a rate of 100–120 per min. Mechanical ventilation was implemented with a tidal volume of 7 ml/kg, FiO_2_ of 1.0 and a rate of 10 breaths per min. After 2 min of CPR, the first bolus of epinephrine was given at a dose of 20 μg/kg, followed by the same dose of epinephrine administration at an interval of 4 min. After 8 min of CPR, a single 150-J biphasic electrical shock was delivered by the M Series Monitor Defibrillator. If an organized rhythm with a mean arterial pressure (MAP) > 50 mm Hg persisted for 5 min or more, the animal was regarded as successful resuscitation. If not, CPR were immediately resumed for 2 min prior to another electrical shock. This protocol was repeated until successful resuscitation or for a total of 18 min. When a recurrent VF occurred after resuscitation, a 150-J biphasic electrical shock was attempted. Following successful resuscitation, mechanical ventilation was continued and the anesthesia was maintained for 4 h. Subsequently, all catheters were removed and the wounds were surgically sutured. The endotracheal tube was removed when the animal recovered from the anesthesia and had spontaneous respiration. The animals were then sent back to their cages for an additional 20 h of observation, in which they had free access to water and food and furthermore obtained the prevention of infection by intramuscular injection of cefotiam (1.0 g) at 4 and 16 h post-resuscitation. At the end of 24 h post-resuscitation, the animals were euthanized with an intravenous injection of propofol (3 mg/kg) and then 10 ml of potassium chloride (10 mol/L). Thereafter, a necropsy was routinely performed to record those possible injuries to the bony thorax and thoracic and abdominal viscera resulting from the surgical or CPR intervention or the presence of obfuscating diseases.

### Measurements

Hemodynamics, electrocardiogram and pulse oxygen saturation were continuously recorded by a patient monitoring system (BeneVision N22, Mindray, Shenzhen, China). Arterial blood gas and lactate concentrations were measured at baseline, 1, 2, and 4 h after resuscitation on 2 ml of arterial blood samples with a Blood Gas Analyzer (iSTAT300, Abbott, Chicago, United States).

Cardiac function, indicated by the changes of SV and GEF, was evaluated with the PiCCO system at baseline, 1, 2, and 4 h after resuscitation. Cardiac and cerebral injury biomarkers including cardiac troponin I (cTnI), neuron specific enolase (NSE) and S100B protein (S100B) were measured at baseline, 1, 2, 4, and 24 h after resuscitation, in which venous blood samples were collected and then the serums were obtained for their measurements with enzyme-linked immunosorbent assay (ELISA) kits (Meixuan Biotechnology Inc, Shanghai, China) according to the manufacturer’s instructions. Cerebral function was evaluated based on the method of neurological deficit score (NDS) at 24 h after resuscitation, in which the NDS was scored from 0 (no observed neurologic deficit) to 400 (death or brain death) (Berg et al., 1994), and examined by two investigators who were blinded to the study.

The distribution of MSCs in the heart and brain at 24 h after resuscitation was detected by immunofluorescence staining according to the manufacturer’s instructions. First, left ventricular myocardium, and left cortex and hippocampus were harvested immediately after animal euthanasia, then fixed in 4% paraformaldehyde for 24 h, thereafter embeded in paraffin, and finally cut in a 5-µm section. Subsequently, the specimens were incubated with anti-CD73 (1:100, Abcam, Cambridge, MA), anti-CD90 (1:100, Abcam, Cambridge, MA), and anti-CD105 (1:100, Abcam, Cambridge, MA) at 4°C for 24 h, then incubated with secondary antibody for 1 h, and finally stained with DAPI for 10 min. After that, the fluorescence of CD73, CD90 and CD105 protein adducts and DAPI were observed with the fluorescence microscope. For the measurement of cardiac and cerebral apoptosis at 24 h after resuscitation, the specimens were similarly obtained, and then stained using the TdT-mediated dUTP nick end labelling (TUNEL) assay kit (Boster Biological Technology co, Wuhan, China) according to the manufacturer’s instructions. After that, at least six views were randomly chosen to count the numbers of TUNEL-positive cells and total cells at ×200 magnification under an optical microscope (Biological microscope C×31, Olympus, Japan). The rate of apoptotic cells was calculated as the percentage of TUNEL-positive cells/total cells.

Pyroptosis-related proteins including NOD-like receptor protein 3 (NLRP3), apoptosis-associated speck-like protein containing a caspase recruitment domain (ASC), cleaved caspase-1 and gasdermin D (GSDMD), and ferroptosis-related proteins including acyl-CoA synthetase long-chain family member 4 (ACSL4) and glutathione peroxidase 4 (GPX4) in the heart and brain at 24 h after resuscitation were detected by Western blot. First, tissue specimens in the left ventricular myocardium, and right cortex and hippocampus were similarly harvested after animal euthanasia. Second, tissue protein was extracted with a RIPA lysate containing a protease inhibitor, then centrifuged at 12, 000 rpm at 4°C for 20 min, and finally quantified by a BCA protein quantitation kit (Beyotime Biotechnology, Shanghai, China). Third, a weight of 40 μg of protein sample was separated by 10% SDS-PAGE, then transferred to a PVDF membrane, and finally blocked in 5% non-fat milk diluted by TBST solution. Fourth, all the membranes were incubated with primary anti-NLRP3 (1:1,000, Proteintech, Rosemount, United States), anti-ASC (1:1,000, Proteintech, Rosemount, United States), anti-cleaved caspase-1 (1:1,000, Cell Signaling Technology, Danvers, United States), anti-GSDMD (1:1,000, Proteintech, Rosemount, United States), anti-ACSL4 (1:1,000, Proteintech, Rosemount, United States), anti-GPX4 (1:1,000, Proteintech, Rosemount, United States) and anti-glyceraldehyde-3-phosphate dehydrogenase (GAPDH) (1:1,000, BBI Life Science Corporation, Shanghai, China) at 4°C for 24 h, then rinsed with TBST solution, and finally incubated with the secondary antibody (1:5,000, BBI Life Science Corporation, Shanghai, China) at room temperature for 1 h. The protein was visualized with ECL luminescent substrates, and then analyzed by ImageJ software (NIH, Bethesda, United States). The expression levels of NLRP3, ASC, cleaved caspase-1, GSDMD, ACSL4 and GPX4 were all standardized by GAPDH.

The contents of proinflammatory cytokines including interleukin-1β (IL-1β) and interleukin-18 (IL-18) in the heart and brain at 24 h after resuscitation were measured using the ELISA kits (Meixuan Biotechnology Inc, Shanghai, China). After cardiac and cerebral tissue specimens were harvested, they were homogenized with normal saline on ice and then centrifuged at 4,000 rpm at 4°C for 15 min. After that, the supernatants were collected for measuring the levels of IL-1β and IL-18 according to the manufacturer’s instructions. The level of iron deposition in the heart and brain at 24 h after resuscitation was measured by Prussian blue staining. Cardiac and cerebral tissue specimens were similarly harvested, and then fixed in 4% paraformaldehyde, embeded in paraffin, and finally cut in a 5-µm section. Thereafter, the sections were rehydrated, incubated with Prussian blue staining solution, rinsed in distilled water, and stained with nuclear fast red. Finally, the Prussian blue-stained specimens were photographed at ×200 magnification under an optical microscope, and then the percentage of positive staining area was analyzed.

### Statistical Analysis

All data were analyzed using the SPSS 20.0 statistical software (IBM, Armonk, United States). The distribution of continuous variables was confirmed with the Kolmogorov-Smirnov test. After the data were confirmed to be normally distributed, they were presented as mean ± standard deviation and compared with one way analysis of variance (ANOVA). Comparisons between time-based measurements within each group were performed with repeated-measurement analysis of variance. If there was a significant difference in the overall comparison of groups, comparisons between any other two groups were made by the Bonferroni test. For the comparison of categorical variables such as the rates of resuscitation success and 24-h survival, the data were compared with the Fisher’s exact test. A value of *p* < 0.05 was considered significant.

## Results

**FIGURE 2:**
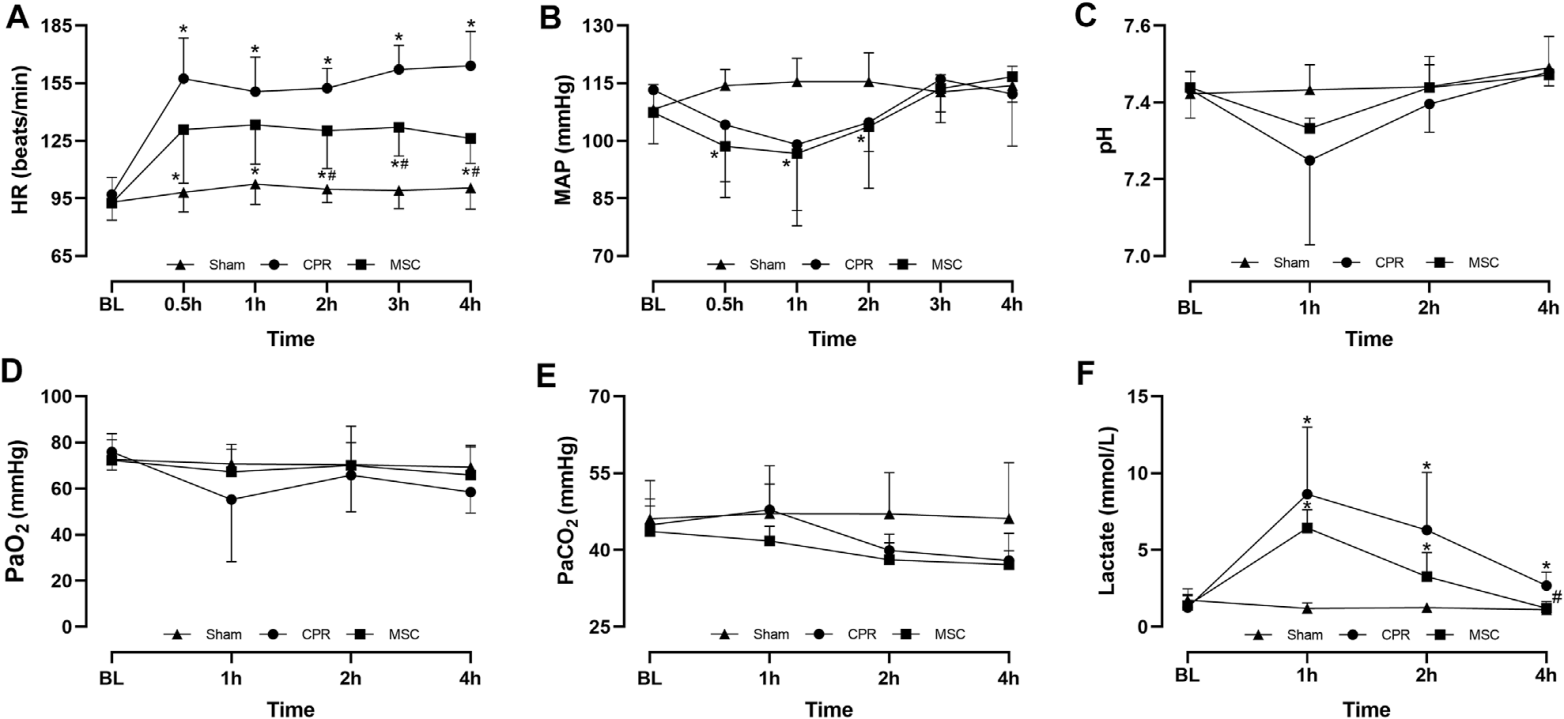
Hemodynamics, blood gas and lactate **(A)** Heart rate (HR) **(B)** Mean arterial pressure (MAP) **(C)** pH **(D)** PaO_2_ (**E**) PaCO_2_ **(F)** Lactate. BL, baseline; CPR, cardiopulmonary resuscitation; MSC, mesenchymal stem cell. ^*^ *p* < 0.05 versus sham group; ^#^ *p* < 0.05 versus CPR group.

**FIGURE 3:**
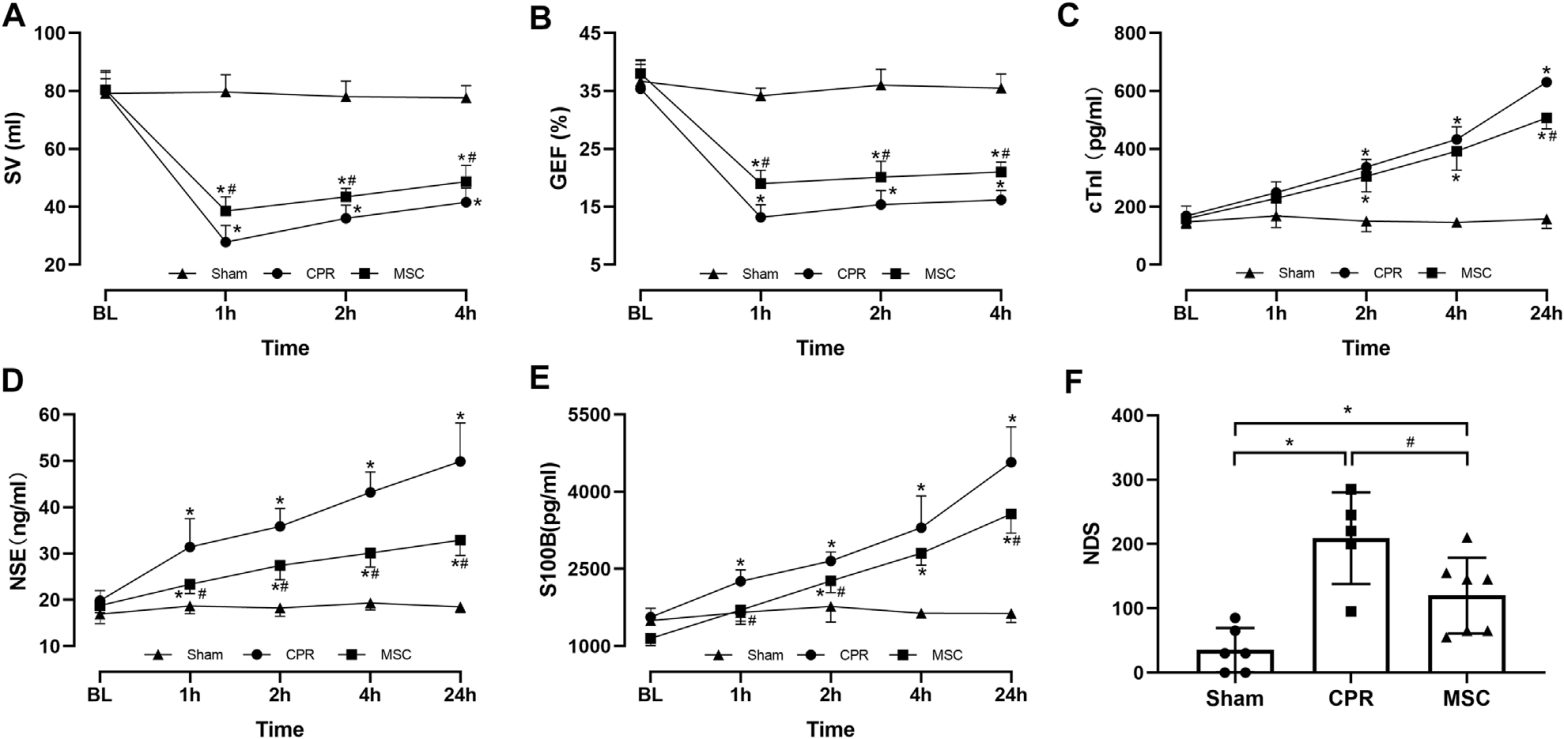
Cardiac, cerebral function and injury biomarkers **(A)** Stroke volume (SV) **(B)** Global ejection fraction (GEF) **(C)** Cardiac troponin I (cTnI) **(D** Neuron specific enolase (NSE) **(E)** S100B protein (S100B) **(F)** Neurological deficit score (NDS). BL, baseline; CPR, cardiopulmonary resuscitation; MSC, mesenchymal stem cell. ^*^ *p* < 0.05 versus sham group; ^#^ *p* < 0.05 versus CPR group.

Twenty-four experiments were performed and completed in this study. Before CA, baseline measurements including hemodynamics, blood gas, lactate, and cardiac, cerebral function and injury biomarkers were not significant among the three groups (Figures 2, 3). During CPR, five of the ten animals in the CPR group and seven of the eight animals in the MSC group were successfully resuscitated, in which the rate of resuscitation success was not significant between the two groups (*p* = 0.152). In the following period of post-resuscitation observation, all resuscitated animals survived for 24 h, and the difference in the 24-h survival rate was not significant between the two groups (*p* = 0.152).

During post-resuscitation observation, HR was higher and MAP was lower in all resuscitated animals compared with the sham group; however, a slower increase in HR was observed in the MSC group than in the CPR group, in which the differences were significant starting 2 h after resuscitation between the two groups. In addition, MAP was decreased to an even physiological level of greater than 97 mmHg and then returned to a near-baseline level in the two groups (Figures 2A, B). Post-resuscitation pH was lower and lactate was higher in all resuscitated animals compared to the sham group; however, a faster improvement in them was observed in the MSC group, in which the value of lactate at 4 h post-resuscitation was significant lower than that in the CPR group. There were no differences in arterial PO_2_ and PCO_2_ throughout the experiment among the three groups (Figures 2C–F).

Post-resuscitation SV and GEF were significantly lower and cTnI was significantly higher in the CPR and MSC groups compared with the sham group. However, the values of SV and GEF were significantly greater in the MSC group than in the CPR group. In addition, the serum levels of cTnI after resuscitation were always lower in the MSC group than in the CPR group, in which the difference was significant at 24 h post-resuscitation between the two groups (Figures 3A–C). Similarly, post-resuscitation NSE and S100B were significantly higher in the CPR and MSC groups compared to the sham group. However, the serum levels of NSE at all the time points after resuscitation and the serum levels of S100B at 1, 2 and 24 h after resuscitation were significantly lower in the MSC group when compared with the CPR group. In addition, post-resuscitation cerebral function evaluated by NDS was significantly impaired in the CPR and MSC groups compared to the sham group. However, cerebral function was significantly better in the animals receiving the MSCs compared to the CPR group (Figures 3D–F).

**FIGURE 4:**
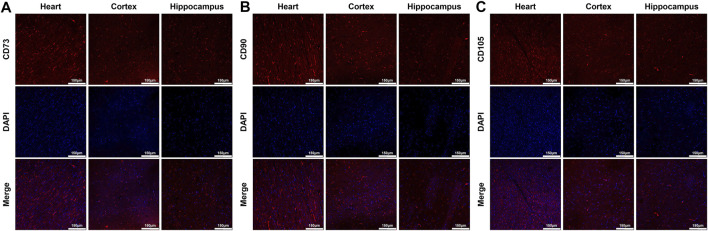
Distribution of MSCs in the heart, cortex and hippocampus **(A)**–**(C)** Representative photomicrographs of immunofluorescence staining of MSCs’ surface markers CD73, CD90 and CD105 (×200magnification). MSC, mesenchymal stem cell.

**FIGURE 5:**
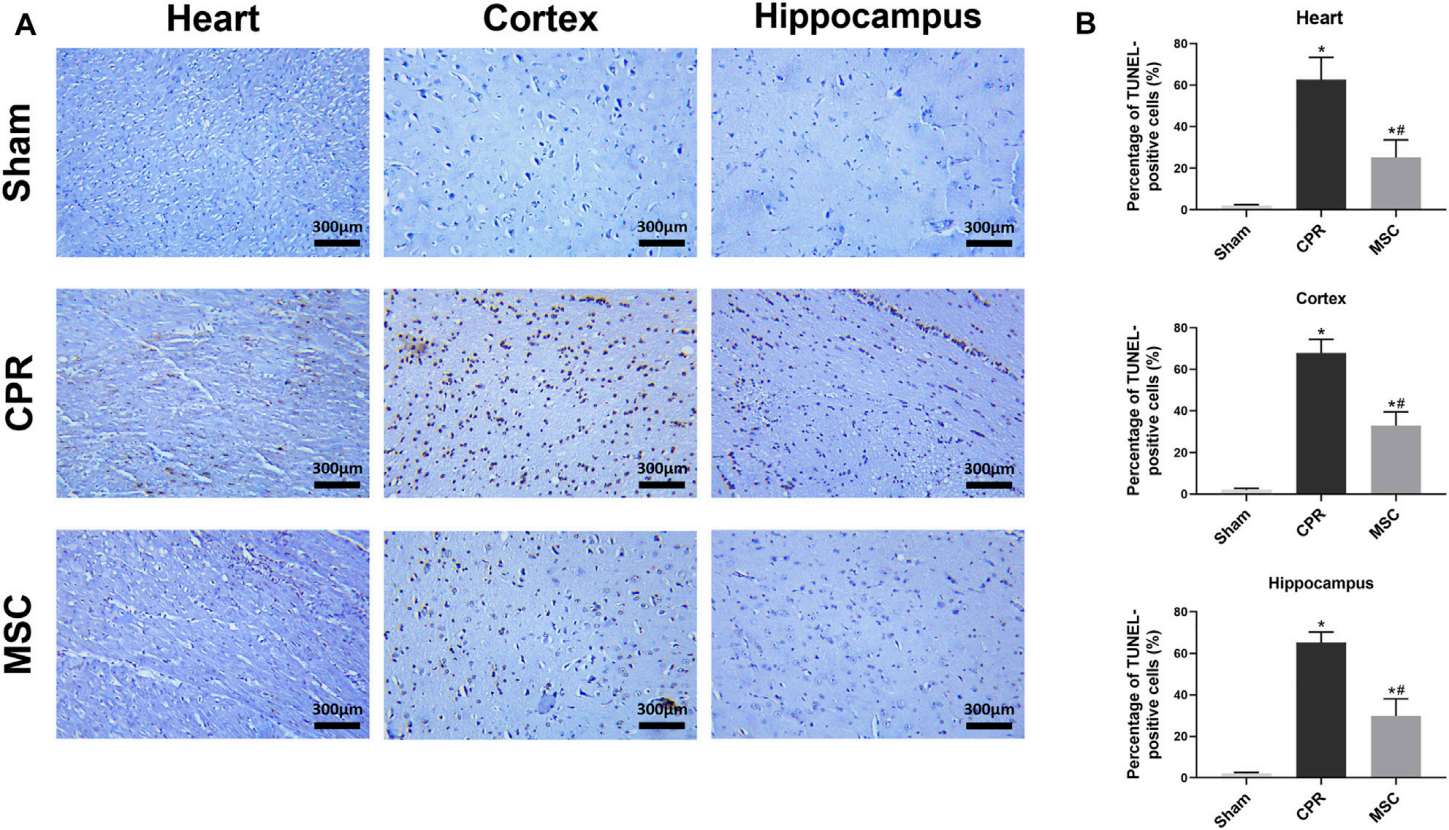
Cell apoptosis in the heart, cortex and hippocampus **(A)** Representative photomicrographs of TUNEL assay (×200magnification) **(B)** The percentage of TUNEL-positive cells. TUNEL, TdT-mediated dUTP nick end labelling; CPR, cardiopulmonary resuscitation; MSC, mesenchymal stem cell. ^*^ *p* < 0.05 versus sham group; ^#^ *p* < 0.05 versus CPR group.

At 24 h post-resuscitation, those specific surface markers of MSCs including CD73, CD90 and CD105 were successfully detected by immunofluorescence staining in the heart, cortex and hippocampus in the survived animals in the MSC group, which indicated the anchorage of MSCs into these areas through the network of blood vessels (Figure 4). In addition, cell apoptosis evaluated by the percentage of TUNEL-positive cells were significantly higher in the heart, cortex and hippocampus in all animals experiencing CA and resuscitation compared with the sham group. However, the administration of MSCs significantly decreased cardiac and cerebral apoptosis when compared to the CPR group (Figures 5A,B).

**FIGURE 6:**
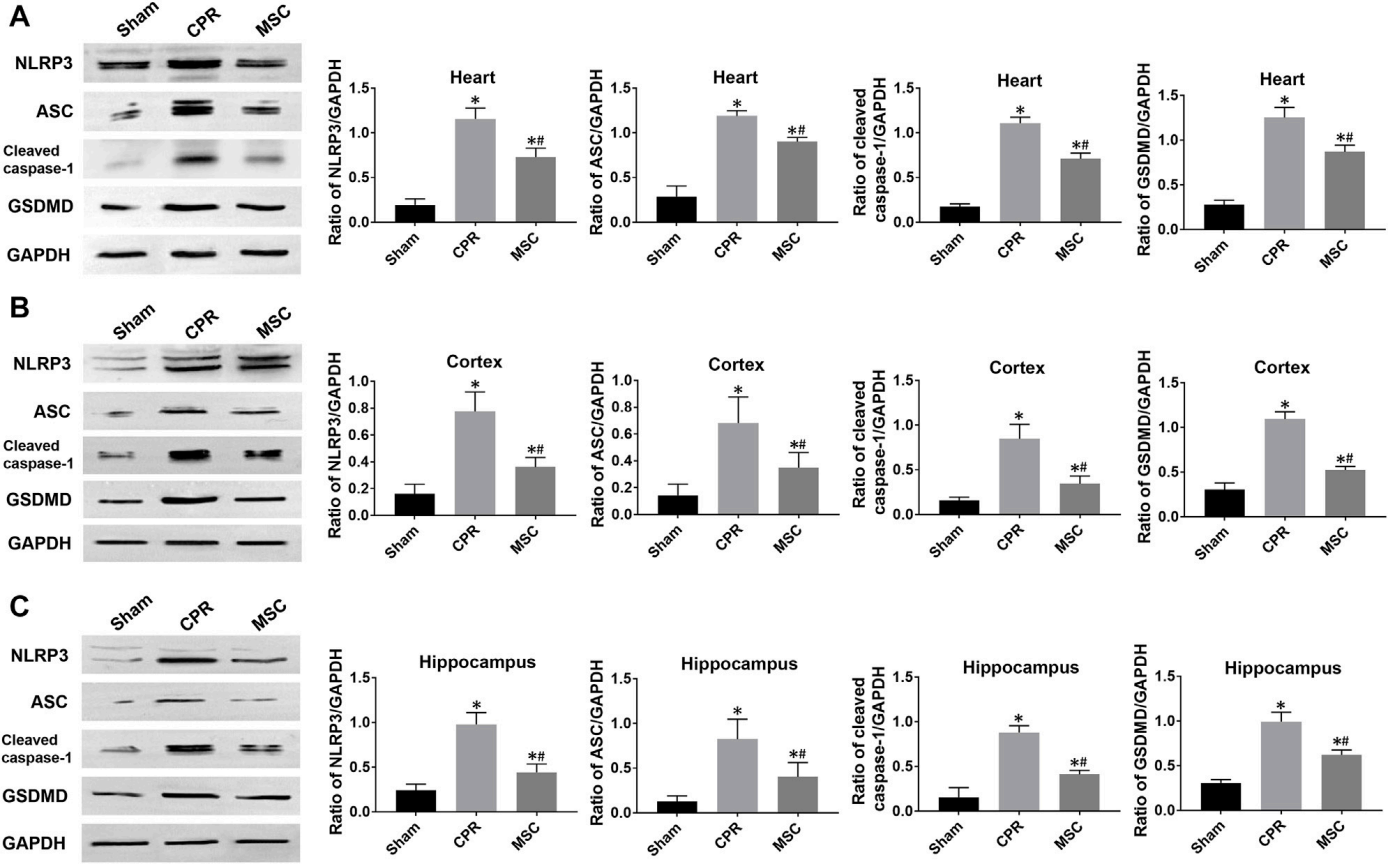
Expression of pyroptosis-related proteins **(A)**–**(C)** The expression levels of NLRP3, ASC, cleaved caspase-1 and GSDMD in the heart, cortex and hippocampus. NLRP3, NOD-like receptor protein 3; ASC, apoptosis-associated speck-like protein containing a caspase recruitment domain; GSDMD, gasdermin D; CPR, cardiopulmonary resuscitation; MSC, mesenchymal stem cell. ^*^ *p* < 0.05 versus sham group; ^#^ *p* < 0.05 versus CPR group.

**FIGURE 7:**
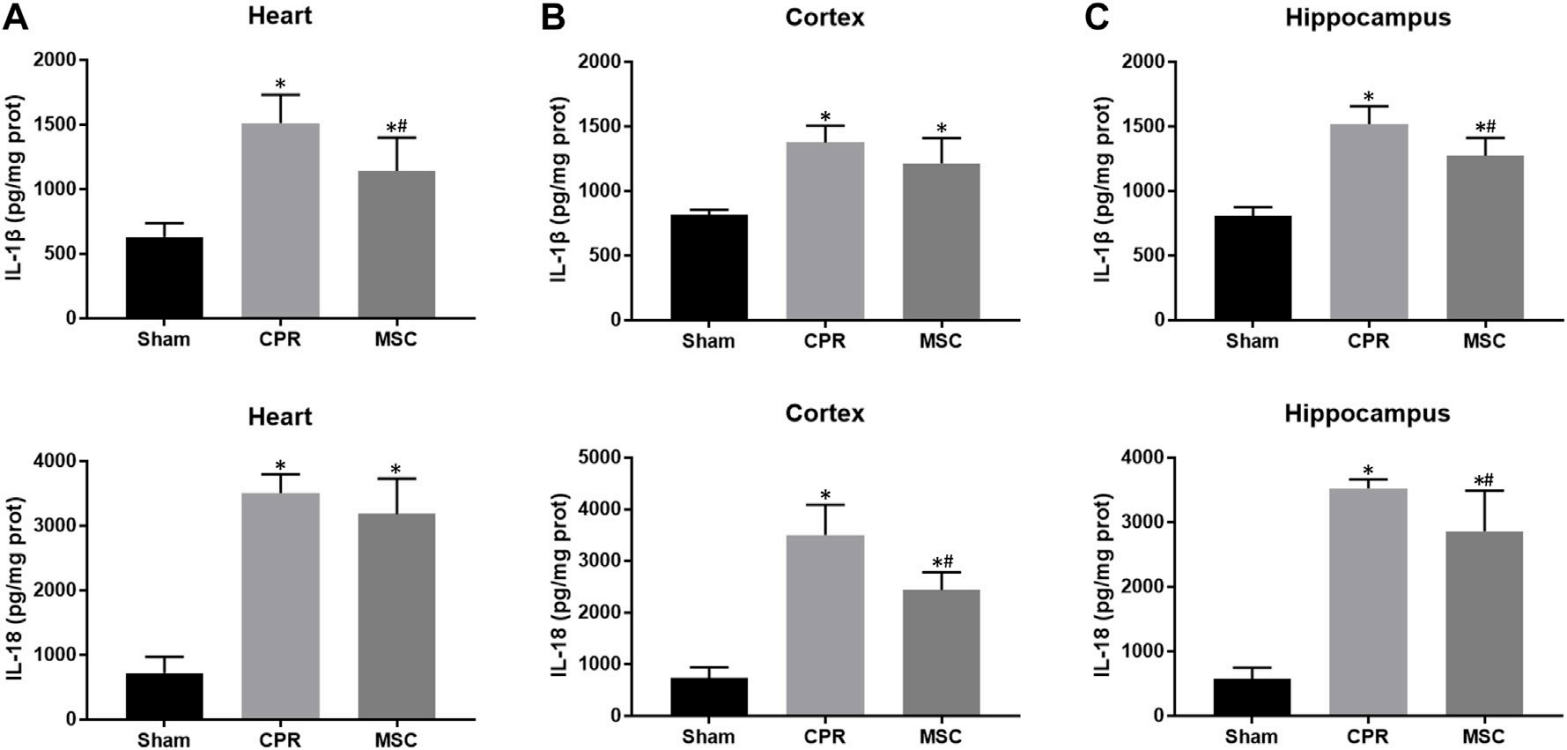
Contents of proinflammatory cytokines **(A)**–**(C)** The contents of IL-1β and IL-18 in the heart, cortex and hippocampus. IL-1β, interleukin-1β; IL-18, interleukin-18; CPR, cardiopulmonary resuscitation; MSC, mesenchymal stem cell. ^*^ *p* < 0.05 versus sham group; ^#^ *p* < 0.05 versus CPR group.

At 24 h post-resuscitation, the expression levels of pyroptosis-related proteins including NLRP3, ASC, cleaved caspase-1 and GSDMD were significantly higher in the heart, cortex and hippocampus in all animals experiencing CA and resuscitation when compared with the sham group. However, all of them were significantly lower in animals treated with MSCs when compared to the CPR group (Figures 6A–C). Similarly, the contents of proinflammatory cytokines including IL-1β and IL-18 in the heart, cortex and hippocampus at 24 h post-resuscitation were significantly higher in the CPR and MSC groups than in the sham group. Nevertheless, both of them were lower in the MSC group than in the CPR group, in which the contents of IL-1β in the heart and hippocampus, and the contents of IL-18 in the cortex and hippocampus were significantly lower in animals treated with MSCs when compared to the CPR group (Figures 7A–C). Thus, treatment with MSCs significantly inhibited cardiac and cerebral pyroptosis when compared to the CPR group.

**FIGURE 8:**
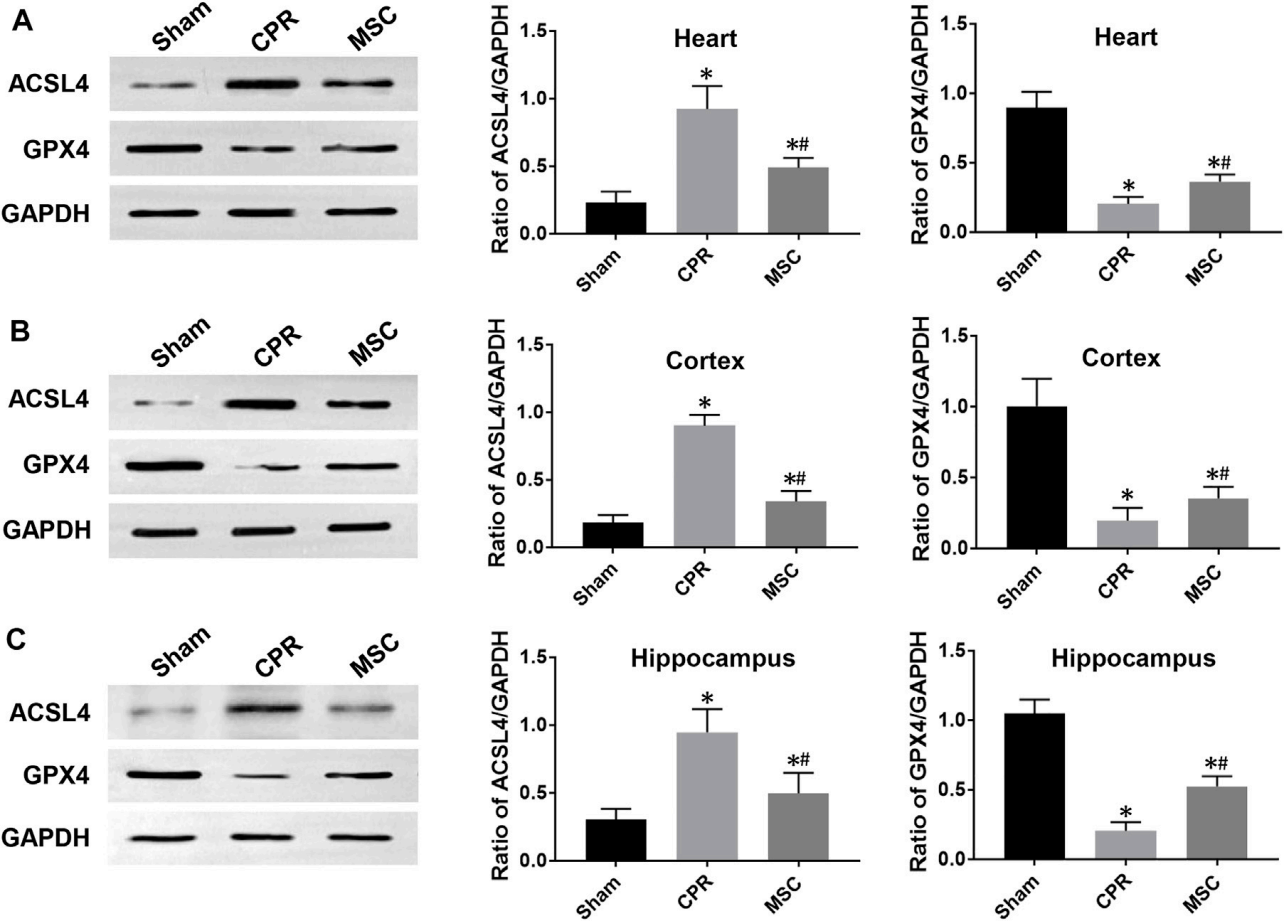
Expression of ferroptosis-related proteins **(A)**–**(C)** The expression levels of ACSL4 and GPX4 in the heart, cortex and hippocampus.ACSL4, acyl-CoA synthetase long-chain family member 4; GPX4, glutathione peroxidase 4; CPR, cardiopulmonary resuscitation; MSC, mesenchymal stem cell. ^*^ *p* < 0.05 versus sham group; ^#^ *p* < 0.05 versus CPR group.

**FIGURE 9:**
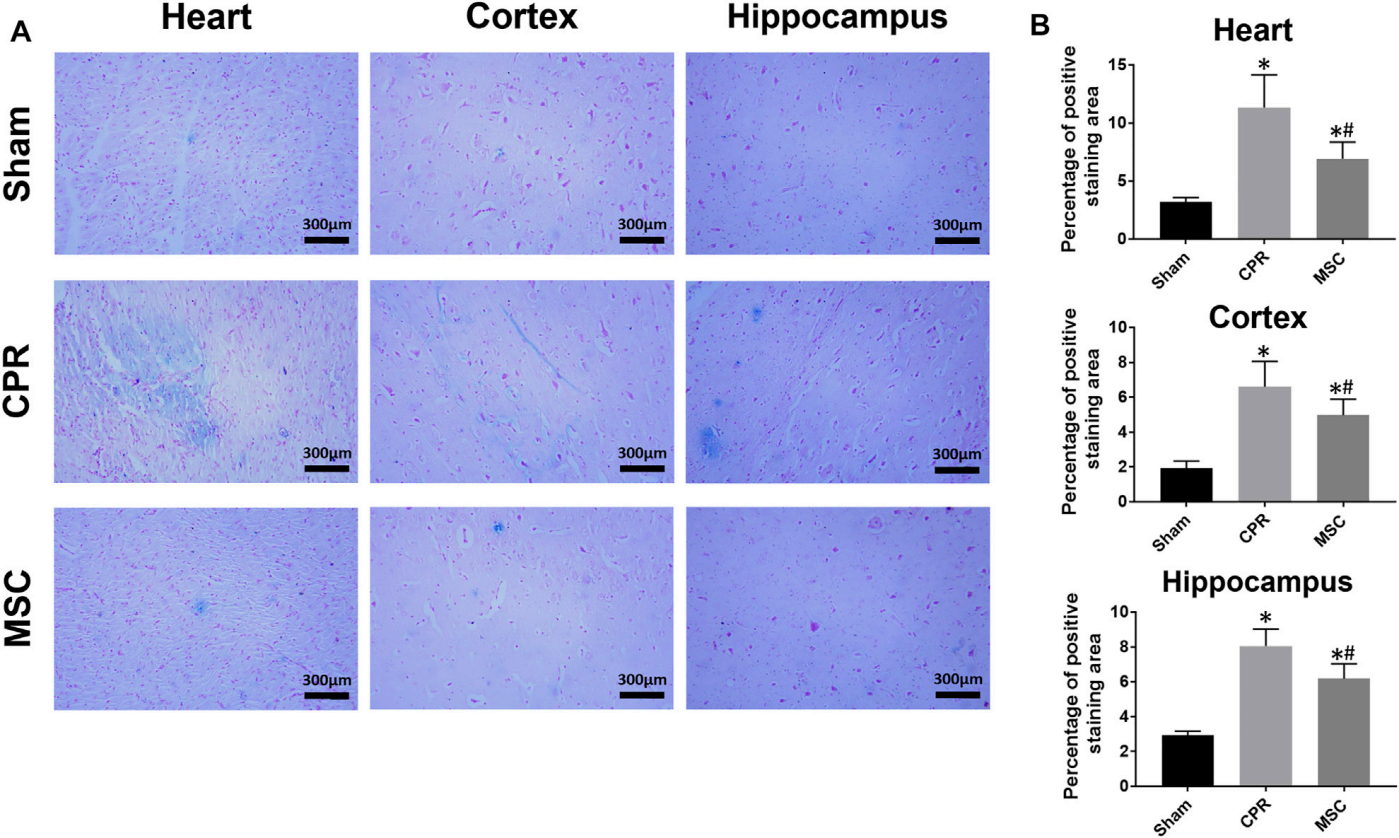
Iron deposition in the heart, cortex and hippocampus **(A)** Representative photomicrographs of Prussian blue staining (×200magnification) **(B)** The percentage of positive staining area. CPR, cardiopulmonary resuscitation; MSC, mesenchymal stem cell. ^*^ *p* < 0.05 versus sham group; ^#^ *p* < 0.05 versus CPR group.

At 24 h post-resuscitation, cell ferroptosis was evaluated by the changes of its protein markers ACSL4 and GPX4 and the level of iron deposition in the heart, cortex and hippocampus. Consequently, ACSL4 expression and iron deposition were significantly higher while GPX4 expression was significantly lower in all animals experiencing CA and resuscitation compared with the sham group. However, in animals treated with MSCs, the increase in ACSL4 expression and iron deposition and the decrease in GPX4 expression were all significantly milder when compared to the CPR group (Figures 8, 9). Thus, cardiac and cerebral ferroptosis were significantly reduced in the animals receiving the MSCs when compared to the CPR group.

## Discussion

The present study employed a clinically relevant, large-animal model and investigated the effects of MSCs administration on cardiac and cerebral outcomes after CA and resuscitation in swine. First, hESCs-derived MSCs were preferentially chosen based on two previous studies (Lv et al., 2012; Wu et al., 2020), in which they showed several advantages over the primary MSCs derived from the umbilical cord or bone marrow, such as a higher quality, stronger immunomodulatory function and greater ability to treat organ injury. Second, several preclinical and clinical studies have demonstrated that a dosage range of (1-10)×10^6^/kg of MSCs was well-tolerated in large animals and patients, in which a higher dose could result in a greater therapeutic effect on acute lung injury and acute respiratory distress syndrome (Asmussen et al., 2014; Wilson et al., 2015; Hashemian et al., 2021), so an intermediate dose of 5×10^6^/kg of MSCs was decided to use in the present study. Third, cardiac and cerebral tissue samples were harvested for the evaluation of cell pyroptosis and ferroptosis at 24 h post-resuscitation according to another three studies, which indicated that these two forms of cell death were obvious at 24 h after acute cardiac and cerebral injuries such as myocardial infarction, subarachnoid hemorrhage, and intracerebral hemorrhage (Zhang et al., 2018; Liu W et al., 2021; Xu P et al., 2021). We demonstrated that the administration of MSCs significantly improved post-resuscitation hemodynamic abnormality and tissue hypoperfusion, and markedly alleviated post-resuscitation cardiac, and cerebral dysfunction and injuries compared with the CPR group. In addition, several forms of cell death including apoptosis, pyroptosis and ferroptosis were observed in the heart and brain after resuscitation; however, treatment with MSCs significantly decreased cardiac, cerebral apoptosis, pyroptosis and ferroptosis compared to the CPR group.

To date, the clinical outcomes of CA patients remain dismal, in which the high morbidity and mortality are mainly attributed to severe cardiac and cerebral injuries following successful resuscitation (Laver et al., 2004; Neumar et al., 2008). Despite a series of novel therapeutic interventions including exenatide, erythropoietin and inhaled xenon have been proven to provide potent cardiac and cerebral protection in various experimental models; however, they failed to improve neurological outcome and the survival after CA and resuscitation in the clinical setting (Cariou et al., 2016; Laitio et al., 2016; Wiberg et al., 2016). Even though therapeutic hypothermia has been recommended by the latest CPR guideline to use as an effective treatment for post-resuscitation CA patients (Panchal et al., 2020); nevertheless, a recent open-label trial demonstrated that in those patients treated with therapeutic hypothermia, the rates of mortality and severe disability at 6 m were up to 50 and 55%, respectively (Dankiewicz et al., 2021). Currently, one study demonstrated that the administration of MSCs alleviated brain oxidative stress, neuronal cell death and promoted the recovery of behavioral function better than therapeutic hypothermia after global cerebral IR injury in rats (Chung et al., 2017). Since cardiac and cerebral protection induced by MSCs have been observed in several small-animal CA models (Wang et al., 2008; Wang et al., 2009; Zhou et al., 2017), it deserves to further confirm the protective effects of MSCs administration in the larger-animal experimental studies and clinical trials. Thus, we translated the previous small-animal study into a clinically relevant swine model. Consequently, post-resuscitation cardiac, cerebral dysfunction and injuries were significantly improved accompanied with a significant decrease in cardiac and cerebral apoptosis in the animals receiving the MSCs compared to the CPR group. Therefore, MSCs administration may become an effective approach to improve post-resuscitation care.

Recently, there has been increasing evidence that cell pyroptosis plays a key role in the pathogenetic process of heart and brain after experiencing IR injury, which could become a therapeutic target for organ protection (Datta et al., 2020; Lu et al., 2021). Additionally, studies have shown that the activation of NLRP3 inflammasome is one of the main approaches to induce cell pyroptosis, in which the NLRP3 inflammasome complex (NLRP3/ASC/caspase-1) recognizes pathogen- and danger-associated molecular patterns, then results in the activation of caspase-1, subsequently cleaves the proinflammatory cytokines IL-1β and IL-18 and the pyroptotic substrate GSDMD, and finally promotes the formation of plasma membrane pores that mediate cell death and excessive release of IL-1β and IL-18 (Paik et al., 2021). In a rat model of CA and resuscitation, one study has demonstrated that N-acetylcysteine improved post-resuscitation myocardial dysfunction and the survival through the alleviation of NLRP3 inflammasome-induced cell pyroptosis (He et al., 2020). In addition, another study has demonstrated that the inhibition of NLRP3 inflammasome activation reduced microglial pyroptosis and furthermore improved neuropathological damage, neurologic outcome and neurocognitive function after resuscitation in rats (Chang et al., 2020). Likewise, cell pyroptosis mediated by NLRP3 inflammasome was observed in the heart and brain after resuscitation in this swine model; however, it was significantly alleviated in animals treated with MSCs compared to the CPR group. Thus, MSCs administration could produce post-resuscitation cardiac and cerebral protection through the inhibition of NLRP3 inflammasome-mediated cell pyroptosis.

Currently, cell ferroptosis emerged as a new type of regulated cell death driven by iron overload and lipid peroxidation, has been linked to various human diseases (Xu S et al., 2021). In the case of regional IR injury, a growing number of studies have also confirmed that cell ferroptosis is involved in the pathogenesis of cardio-cerebral vascular diseases (Liu et al., 2020; Wu et al., 2021). In 2021, one study demonstrated that the activation of divalent metal transporter 1 after acute myocardial infarction promoted cell ferroptosis and thereby resulted in myocardial injury (Song et al., 2021). Another study demonstrated that ACSL4 overexpression exacerbated ischemic stroke through strengthening cell ferroptosis-induced brain injury and neuroinflammation in mice (Cui et al., 2021). However, almost no investigation has reported the phenomenon of cell ferroptosis and its potential influence in the setting of CA and resuscitation. In the present study, we demonstrated that cell ferroptosis occurred in cardiac and cerebral tissues after CA and resuscitation. In addition, the administration of MSCs could decrease cell ferroptosis so as to improve post-resuscitation cardiac and cerebral outcomes. Thus, cell ferroptosis might become an effective therapeutic target for cardiac and cerebral protection after resuscitation.

Although the mechanism by which MSCs regulate post-resuscitation cardiac, cerebral pyroptosis and ferroptosis in a CA model remains unclear; however, several studies have manifested that MSCs alleviate cell pyroptosis and ferroptosis of these organs through multiple approaches in the models of regional IR injury. In the setting of myocardial IR injury, one study demonstrated that MSC-derived exosomes inhibited cardiomyocytes pyroptosis through the regulation of miRNA-100-5p/forkhead box O3/NLRP3 signaling pathway after hypoxia/reoxygenation injury (Liang et al., 2021). Another two studies demonstrated that MSC-derived exosomes attenuated myocardial injury by inhibiting cardiac ferroptosis through promoting miR-23a-3p-mediated suppression of divalent metal transporter 1 expression and modulating the pumilio RNA binding family member 2/peroxiredoxin 6 axis, respectively (Song et al., 2021; Zhang et al., 2021). In the setting of cerebral IR injury, one study demonstrated that MSC-derived exosomes decreased microglial pyroptosis by enhancing forkhead box O3a-mediated mitophagy and subsequently alleviated neuronal injury under hypoxia/reoxygenation (Hu et al., 2021). Another one study demonstrated that MSC-derived exosomes alleviated cerebral I/R injury by inhibiting neuron pyroptosis through modulating microglial polarization (Liu X et al., 2021). However, almost no investigation has reported the regulatory mechanism of MSCs in cerebral ferroptosis after I/R injury.

Our study has some limitations. First, this study was initially designed to investigate the potential benefit of MSCs for those high-risk individuals of CA events. Since a longer time is usually needed to initiate the CPR procedure after experiencing CA events, MSCs pretreatment might provide an additional protection against global IR injury in these high-risk individuals. However, more CA events are unpredictable in the clinical setting, so the effectiveness of MSCs administered during CPR or after resuscitation should be worthy of further exploration in the future. Second, although MSCs administration decreased post-resuscitation cardiac, cerebral pyroptosis and ferroptosis in this swine model of CA; however, the detection of cell pyroptosis and ferroptosis were performed by only some classical indicators using those regular methods. Thus, the changes of cell morphology and more specific proteins, and the potential modulation mechanism related to cell pyroptosis and ferroptosis require further investigation. Third, post-resuscitation cardiac, cerebral function and injuries were only evaluated for 24 h of observation period. A longer observation period is needed to fully evaluate cardiac and cerebral outcomes so as to further confirm the protective effects of MSCs in the following studies.

## Conclusion

In this clinically relevant, large-animal swine model, MSCs administration significantly alleviated post-resuscitation cardiac and cerebral injuries and therefore promoted their functional recovery, in which the protective effects were related to the inhibition of cell pyroptosis and ferroptosis.

## Data Availability

The original contributions presented in the study are included in the article/Supplementary Material, further inquiries can be directed to the corresponding author.
